# Recurrence Patterns and Survival Outcomes in Clinical Stage IIB/IIC Melanoma: Can We Stratify Patients for Consideration of Neoadjuvant Immunotherapy?

**DOI:** 10.1245/s10434-025-18263-z

**Published:** 2025-09-04

**Authors:** Mohammad Saad Farooq, Valentina Mattfeld, Pamela Chopra Beniwal, Gracia Maria Vargas, Neha Shafique, John T. Miura, Giorgos C. Karakousis

**Affiliations:** 1 Division of Endocrine and Oncologic Surgery, Department of Surgery, Hospital of the University of Pennsylvania, Philadelphia, PA USA; 2 Perelman School of Medicine, University of Pennsylvania, Philadelphia, PA USA

**Keywords:** Clinical stage IIB/IIC melanoma, High-risk stage II melanoma, Neoadjuvant immunotherapy, Recurrence, Recurrence-free survival, NCT03757689, Lymphovascular invasion

## Abstract

**Background:**

Interest in evaluating neoadjuvant immunotherapy for stage IIB/IIC melanoma is growing, but studies assessing long-term outcomes generally report data based on pathologic stage after sentinel lymph node microstaging. This study therefore aimed to characterize real-world recurrence patterns and survival specifically in clinical stage IIB/IIC melanoma to contextualize outcomes for selection of patients to undergo neoadjuvant immunotherapy.

**Methods:**

This single-institution retrospective cohort study included patients who received a diagnosis of American Joint Committee on Cancer eighth-edition clinical stage IIB/IIC cutaneous melanoma from 2006 to 2019. Factors associated with recurrence were analyzed using univariable analysis and multivariable Cox proportional hazards analysis. Recurrence-free survival (RFS), melanoma-specific survival (MSS), and overall survival (OS) were analyzed using the Kaplan–Meier method.

**Results:**

The inclusion criteria were met by 229 patients, of whom 152 (66%) were male and 208 (91%) were white. The median follow-up time was 64 months (interquartile range [IQR] 29–105 months). Overall, 101 (44%) patients experienced a recurrence, with a median time-to-recurrence of 15.3 months (IQR 8–31 months). The estimated 2-year RFS, MSS, and OS were 69%, 91%, and 87%, respectively. The presence of pre-surgical lymphovascular invasion (hazard ratio [HR] 1.764; *p* = 0.018) was associated with increased risk of recurrence.

**Conclusion:**

This single-institution retrospective cohort study of patients with clinical stage IIB/IIC melanoma found a 2-year RFS of 69%. The presence of pre-surgical lymphovascular invasion was significantly associated with recurrence in this population. These data can help guide clinicians and researchers in the design and assessment of future studies evaluating neoadjuvant therapy in clinical stage IIB/IIC melanoma and in optimizing patient selection.

Cutaneous melanoma contributes to 5% of all new cancer diagnoses per year and 65% of all skin cancer-related deaths.^1–3^ Importantly, although early-stage melanoma can be treated successfully with surgery alone, more advanced stages portend worse recurrence and survival rates.^4^ High-risk stage II melanoma is a particularly aggressive subset, defined as clinical stage IIB for lesions that have a Breslow thickness of 2–4 mm with ulceration (T3b) or a thickness greater than 4 mm without ulceration (T4a), or as clinical stage IIC for lesions greater than 4 mm with ulceration (T4b), but no evidence of nodal disease.^5^

Notably, the survival outcomes for pathologic stage IIB/IIC melanoma are worse than for stage IIIA melanoma or rival stage IIIB melanoma despite the lack of lymph node metastases.^6,7^ Given these findings, elucidation of optimal treatment regimens for high-risk stage II melanoma has been a compelling topic of interest.

Recent clinical trials (KEYNOTE-716; Checkmate-76K) have demonstrated the benefit of adjuvant PD-1 inhibitor (αPD-1) therapy specifically for pathologic stage IIB/IIC patients, finding significant improvement in recurrence-free survival (RFS) versus placebo.^8–10^ Because of these findings, adjuvant treatment of stage IIB/IIC melanoma with αPD-1 therapy has been added to the standard of care.^4^ However, although the introduction of adjuvant αPD-1 therapy for stage IIB/IIC melanoma has been promising, these data also show that approximately only 15% of patients benefit from adjuvant immunotherapy, and differentiating between which patients need systemic therapy versus postoperative surveillance is important, particularly due to the potentially morbid immune-related adverse events (irAEs) associated with immunotherapy.^8,9,11^

Additionally, recent literature contains studies that support the usage of neoadjuvant immune checkpoint inhibitor therapy for stage III/IV melanoma with improved RFS for the neoadjuvant cohort versus adjuvant therapy alone. However, to date, the value of this approach for high-risk stage II melanoma has not been well-described.^12–14^

Given this, a growing need exists to identify which patients might benefit from neoadjuvant immunotherapy, and how to assess early on-treatment pathologic/immunologic responses to personalize therapy and improve outcomes for clinical stage IIB/IIC melanoma. The phase 2 clinical trial NCT03757689 is the first to specifically evaluate neoadjuvant αPD-1 therapy (pembrolizumab) in clinical stage IIB/IIC melanoma and has recently completed accrual, with results pending.^15^ Importantly however, current literature evaluating recurrence/survival outcomes for stage IIB/IIC disease is predominantly based on pathologic staging,^5,16–22^ which may not provide the most clinically relevant data for patient selection in the neoadjuvant setting for high-risk stage II melanoma (i.e., certain patients will be upstaged to stage III after sentinel lymph node biopsy [SLNB]). To contextualize the findings from NCT03757689 and other studies that may seek to evaluate neoadjuvant immunotherapy in high-risk stage II disease, we aimed to characterize real-world recurrence patterns and survival outcomes specifically in clinical stage IIB/IIC melanoma.

## Methods

### Study Population

This retrospective single-institution cohort study included patients with a diagnosis of American Joint Committee on Cancer (AJCC) eighth-edition clinical stage IIB/IIC cutaneous melanoma.^6^ Institutional review board approval was obtained before initiation of the study. Patient data were collected from an institutional database of adult patients with melanoma treated at the University of Pennsylvania Health System between 1 January 2006 and 31 December 2019. The overall database population was filtered to include only patients with AJCC clinical tumor stage T3b (> 2–4 mm, ulcerated), T4a (> 4 mm, non-ulcerated), and T4b (> 4 mm, ulcerated) primary tumors on initial biopsy. Clinical stage was specifically chosen to contextualize prognostication of outcome before availability of surgical data.

The study excluded patients with non-melanoma concurrent active malignancy or multiple primary melanomas, as well as patients missing key clinicopathologic variables and those with a follow-up time shorter than 1 month. All data were collected by manual electronic health record review and adjudicated by multiple members of the study team to ensure accuracy.

### Clinicopathologic Variables

Specific patient variables collected included age, sex, race, follow-up time, and whether the patient received SLNB, completion lymph node dissection (CLND) and/or postoperative systemic/regional therapy (adjuvant or post-recurrence). The specific tumor variables collected included Breslow thickness, ulceration, lymphovascular invasion (LVI), tumor-infiltrating lymphocytes (TILs), mitotic rate, regression, histologic type, anatomic location, and sentinel lymph node (SLN) status.

### Outcome Measures

The specific outcome measures of interest included recurrence rate and time-to-recurrence, as well as RFS, overall survival (OS), and MSS at 2- and 5-year intervals. Time zero was defined as the date of primary melanoma wide local excision (WLE). Type of recurrence was defined as local/in-transit, regional nodal, or distant. If multiple types of recurrences were detected simultaneously, the most distant classification was used. Treatment regimens after WLE were characterized specifically for CLND and adjuvant or post-recurrence systemic/regional therapy including immunotherapy, targeted therapy, chemotherapy, radiation therapy, or other systemic/locoregional therapies.

### Statistical Analyses

The overall study population was analyzed using descriptive statistics. For continuous variables, medians with interquartile ranges (IQRs) and/or means with standard deviations were reported. Continuous variables were analyzed using Wilcoxon rank-sum tests and unpaired *t* tests, whereas categorical variables were analyzed with Pearson’s chi-square tests and Fisher’s exact tests as appropriate. Univariable analysis was performed to analyze clinicopathologic factors associated with recurrence.

The Kaplan-Meier (KM) method was used to estimate RFS, OS, and MSS. Univariable analyses of survival between groups were performed using the log-rank test. To further explore factors associated with RFS, univariable KM subgroup analyses by American Joint Committee on Cancer (AJCC) eighth-edition clinical stage, ulceration status, Breslow thickness (**a** 2–4 mm; **b** > 4 mm), LVI status, TIL status, mitotic rate (**a** 0–10; **b** > 10), regression, anatomic location, and SLN status were determined. Additionally, a multivariable Cox proportional hazards model was used to analyze pre-surgical factors associated with recurrence in the overall cohort. The analysis did not include SLN status in the multivariable model as a covariate intentionally because this information is not available in the pre-surgical setting, which would be most relevant to patients being considered for neoadjuvant immunotherapy.

Additionally, a subset KM analysis of RFS was performed for patients stratified by receipt of adjuvant therapy versus no adjuvant therapy. Finally, subset Cox proportional hazards analysis was performed to assess era effects on recurrence. For this analysis, 2015 was used as the cutoff year due to approval of ipilimumab for operable melanoma in the adjuvant setting,^23^ and 2016–2019 was used as the “post” cohort versus 2009–2015 as the “pre” cohort; Cox analysis was performed both by incorporating era as a covariate, and by evaluating clinicopathologic factors within the 2009–2015 and 2016–2019 cohorts individually.

An alpha level of 0.05 was applied to designate statistical significance. All analyses were conducted using Stata software version 17 (StataCorp, College Station, TX, USA).

## Results

### Patient and Disease Characteristics

Of 229 eligible patients, 152 (66%) were male, and 208 (91%) were white. The median age of the overall cohort at diagnosis was 62 years (IQR 53–73 years). In terms of clinical stage, 157 (69%) patients had stage IIB melanoma, whereas 72 (31%) patients had IIC melanoma. The median Breslow thickness was 4 mm (IQR 2.8–5.3 mm), and 196 (86%) patients had ulcerated tumors. Detailed clinicopathologic characteristics of the overall cohort are summarized in Table 1. The median follow-up time for the overall cohort was 64 months (IQR 29–105 months).

**Table 1:** Clinicopathologic characteristics of the overall patient cohort by univariable analysis for patients with recurrence vs those without recurrence

Clinicopathologic factor	Overall (*n* = 229) *n* (%)	No recurrence (*n* = 128) *n* (%)	Recurrence (*n* = 101) *n* (%)	*p* value
**Median age: years (IQR)**	62 (53–73)	64 (54–75)	60 (50–71)	0.107
**Sex**				0.581
Male	152 (66)	83 (65)	69 (68)	
Female	77 (34)	45 (35)	32 (32)	
**Race**				0.127
White	208 (91)	112 (88)	96 (95)	
Black	6 (3)	4 (3)	2 (2)	
Other/unreported	15 (6)	12 (9)	3 (3)	
**Tumor location**				0.464
Extremity	114 (50)	69 (54)	45 (44)	
Head/neck	28 (12)	15 (12)	13 (13)	
Trunk	87 (38)	44 (34)	43 (43)	
**Clinical stage**				0.520
IIB	157 (69)	90 (70)	67 (66)	
IIC	72 (31)	38 (30)	34 (34)	
**Breslow thickness (mm)**				0.900
Median (IQR)	4 (2.8–5.3)	4 (2.7–5.6)	3.9 (3–5)	
Mean ± SD	4.5 ± 2.3	4.5 ± 2.4	4.4 ± 2.3	
**Ulceration**				0.178
Absent	33 (14)	22 (17)	11 (11)	
Present	196 (86)	106 (83)	90 (89)	
**LVI**				**0.005**
Absent	157 (69)	97 (76)	60 (59)	
Present	57 (25)	23 (18)	34 (34)	
Unreported	15 (6)	8 (6)	7 (7)	
**Mitotic rate (%)**				0.084
0–10	132 (58)	80 (63)	52 (51)	
> 10	90 (39)	44 (34)	46 (46)	
Unreported	7 (3)	4 (6)	3 (3)	
Median (IQR)	8 (5–14)	8 (5–13)	9.5 (5–15)	0.186
**Regression**				0.626
Absent	162 (71)	89 (70)	73 (72)	
Present	51 (22)	30 (23)	21 (21)	
Unreported	16 (7)	9 (7)	7 (7)	
**TIL**				0.686
Absent	41 (18)	25 (19)	16 (16)	
Present, non-brisk	156 (68)	87 (68)	69 (68)	
Present, brisk	17 (8)	11 (9)	6 (6)	
Unreported	15 (6)	6 (5)	9 (9)	
**Histology**				0.845
Nodular	102 (45)	57 (45)	45 (45)	
Superficial spreading	76 (33)	45 (35)	31 (31)	
Acral	12 (5)	7 (5)	5 (5)	
Desmoplastic	11 (5)	6 (5)	5 (5)	
Other/unspecified	28 (12)	13 (10)	15 (15)	
**SLN status**				**0.004**
Negative	157 (69)	96 (75)	61 (60)	
Positive	65 (28)	26 (20)	39 (39)	
No SLN biopsy	7 (3)	6 (5)	1 (1)	

*IQR* interquartile range, *SD* standard deviation, *LVI* tumor-infiltrating lymphocyte, *TIL* tumor-infiltrating lymphocyte, *SLN* sentinel lymph node

The univariable analysis of the patients who had recurrence (*n* = 101, 44%) versus those who did not (*n* = 128, 56%) showed recurrence was significantly associated with the presence of LVI (34% vs. 18%; *p* = 0.005) and SLN positivity (39% vs. 20%; *p* = 0.004). The presence of mitotic rate higher than 10 versus 0–10 trended toward significance (46% vs. 34%; *p* = 0.084). The remaining clinicopathologic variables showed no significant difference when stratified by recurrence in the univariable analysis (Table 1).

### Recurrence Rates and Survival Outcomes

In the overall cohort, 101 (44%) patients experienced a recurrence. Of the patients who had a recurrence, 65 (64%) experienced it within 2 years of WLE, whereas 94 (93%) experienced it within 5 years. The median time to recurrence was 15.3 months (IQR 8–31 months; Table 2).

**Table 2:** Recurrence and survival outcomes for the overall cohort

**Recurrence: n (%)**:	
Overall:* n* (%)	101 (44)
2-Year recurrence:* n* (%)	65 (64)
5-Year recurrence:* n* (%)	94 (93)
2-Year RFS: % (95% CI)	69 (62–75)
5-Year RFS: % (95% CI)	54 (47–61)
Median time-to-recurrence: months (IQR)	15.3 (8–31)
**Overall survival**	
2-Year OS: % (95% CI)	87 (82–91)
5-Year OS: % (95% CI)	70 (63–76)
**Melanoma-specific survival**	
2-Year MSS: % (95% CI)	91 (86–94)
5-Year MSS: % (95% CI)	81 (74–86)

*RFS* recurrence-free survival, *CI* confidence interval, *IQR* interquartile range, *OS* overall survival, *MSS* melanoma-specific survival

**Fig. 1:**
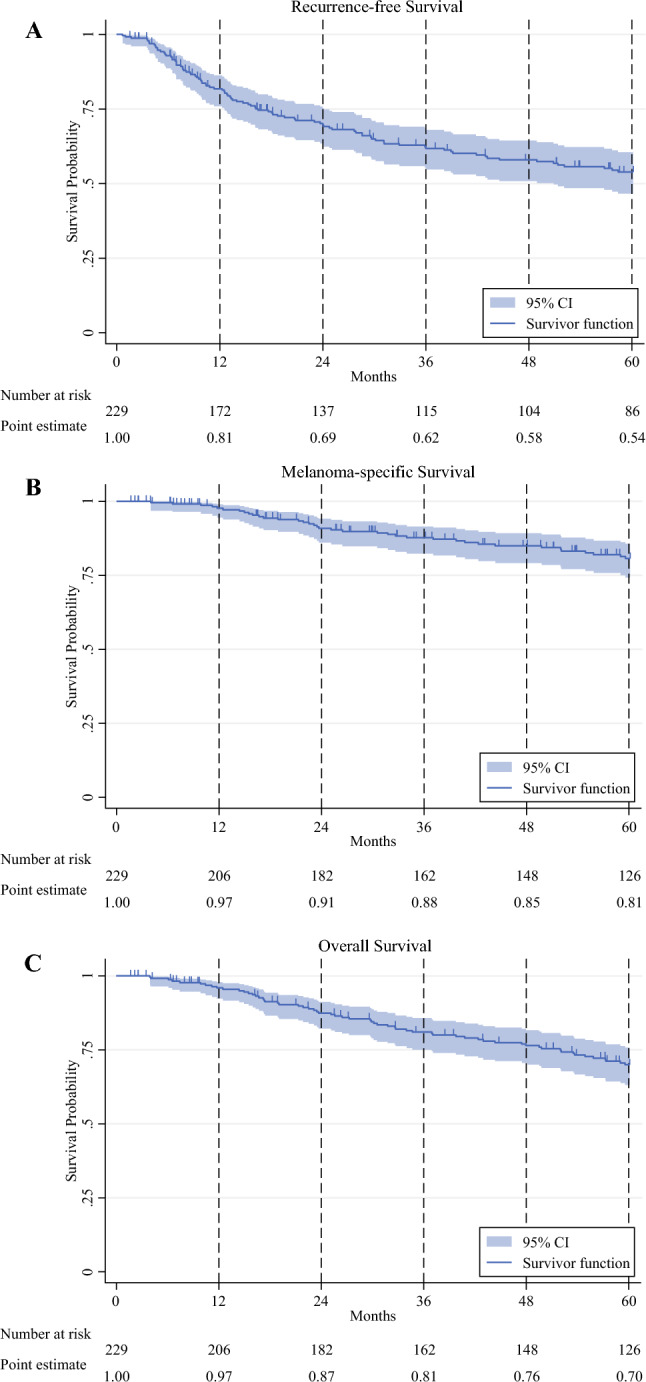
Survival outcomes for the overall cohort during a 5-year period. **A** Recurrence-free survival. **B** Melanoma-specific survival. **C** Overall survival.

**Fig. 2:**
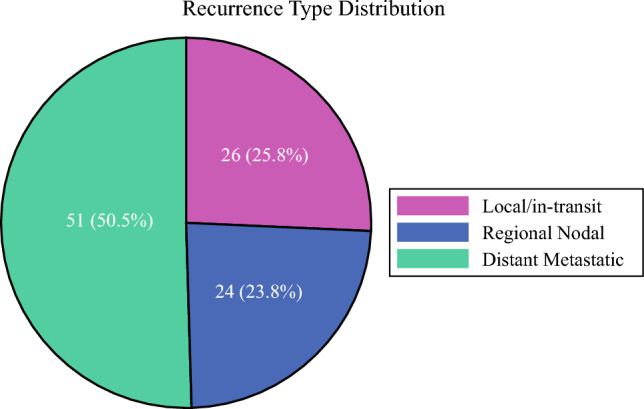
Distribution of recurrence types.

In the KM analysis, the estimated 2-year RFS for the overall cohort was 69% (95% confidence interval [CI] 62–75%; median follow-up time, 37 months [IQR 12–79 months]). The estimated 2-year MSS was 91% (95% CI 86–94%; median follow-up time, 64 months [IQR 30–105 months]), and the 2-year OS was 87% (95% CI 82–91%; median follow-up time, 64 months [IQR 30–105 months]). Detailed survival outcomes are summarized in Table 2 and Fig 1. Of the patients who experienced recurrence, 26 (26%) had local/in-transit, 24 (24%) had regional nodal, and 51 (50.5%) had distant recurrences (Fig. 2).

### Treatment Course

In the overall cohort, 55 (24%) patients went on to receive a CLND, and 101 (44%) received postoperative systemic/regional therapy. Of these patients, 25 (25%) received therapy in the adjuvant setting, whereas 76 (75%) received therapy after recurrence. Detailed postoperative therapy regimens are summarized in Table 3.

**Table 3:** Postoperative treatment course for the overall cohort

	*n* (%)
**Received CLND**	55 (24.0)
**Received postoperative therapy**	101 (44.1)
**Adjuvant**	25 (25)
Immunotherapy	19 (76)
Checkpoint inhibitor	13 (68)
Interferon	3 (16)
GM-CSF (leukine)	4 (21)
Radiation	5 (20)
Targeted therapy	1 (4)
**Post-recurrence**	76 (75)
Immunotherapy	64 (84)
Checkpoint inhibitor	60 (94)
Interferon	3 (5)
IL-2	9 (14)
Radiation	43 (57)
Targeted therapy	28 (37)
Chemotherapy	20 (26)
Other	8 (11)

*CLND* completion lymph node dissection, *GM-CSF* granulocyte macrophage-colony stimulating factor, *IL-2* interleukin-2

### Recurrence-Free Survival Stratified by Pathologic Factor

**Fig. 3:**
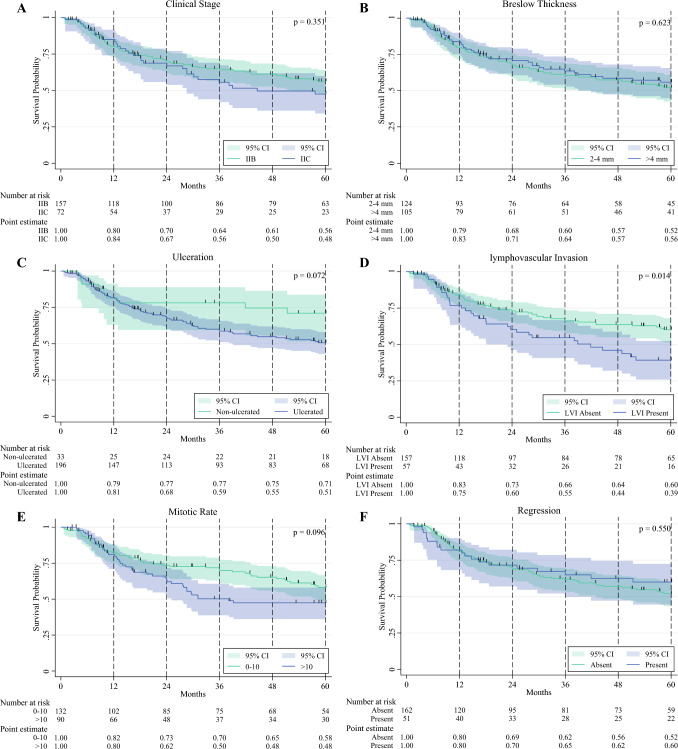

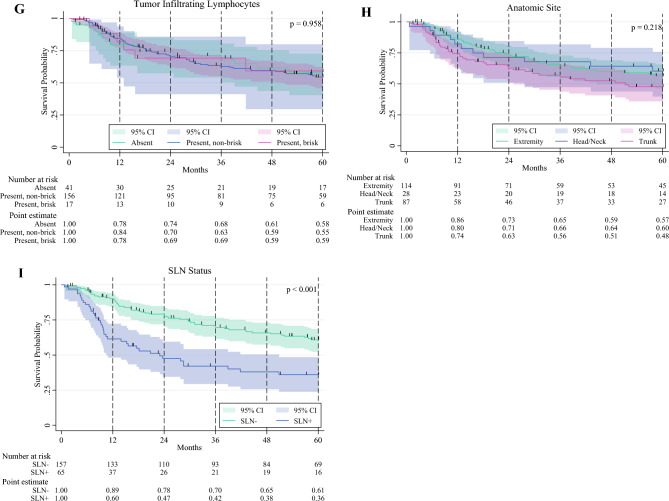
Recurrence-free survival outcomes during a 5-year period stratified by specific pre-surgical pathologic factors. **A** Clinical stage. **B** Breslow thickness. **C** Ulceration. **D** Lymphovascular invasion. **E** Mitotic rate. **F** Regression. **G** Tumor-infiltrating lymphocytes. **H** Anatomic location. **I** Sentinel lymph node status. Log-rank *p* value reported for 5-year timepoint.

In the univariable KM analysis stratified by presence or absence of specific pathologic factors, the presence of LVI was associated with significantly lower estimated 5-year RFS versus absence of LVI (39% vs. 60%; *p* = 0.014). Additionally, positive SLN metastasis also was associated with significantly lower 5-year RFS versus negative SLN (36% vs. 61%; *p* < 0.001). A mitotic rate higher than 10 independently trended toward significance for reduced 5-year RFS versus a mitotic rate of 0-10 (48% vs. 58%; *p* = 0.096), as well as the presence of ulceration versus no ulceration (51% vs. 71%; *p* = 0.072). The remaining stratified pathologic factors (Breslow thickness, regression, TILs, clinical stage, anatomic location) did not demonstrate any significant difference in RFS (Fig. 3).

### Cox Proportional Hazards Analysis of Factors Associated with Recurrence

To further analyze effects of pre-surgical pathologic factors on recurrence, a comprehensive multivariable Cox proportional hazards model was used. The global and individual factor-specific proportional hazards assumption was met. Lymphovascular invasion was found to be independently associated with increased risk for recurrence (hazard ratio [HR] 1.764; 95% CI 1.103–2.819; *p* = 0.018), whereas other pathologic factors did not exhibit a significant association with recurrence (Table 4).

**Table 4:** Multivariable Cox proportional hazards model of pre-surgical factors associated with recurrence

Factor	HR	95% CI	*p* value
**Age (years**)			
< 60	1 (base)		
60+	0.703	(0.457–1.084)	0.110
**Sex**			
Female	1 (base)		
Male	1.219	(0.740–2.006)	0.436
**Race**			
White	1 (base)		
Non-white	Non-estimable	–	–
**Tumor location**			
Extremity	1 (base)		
Head/neck	1.168	(0.557–2.451)	0.681
Trunk	1.361	(0.836–2.217)	0.216
**Breslow thickness (mm)**			
> 2–4	1 (base)		
> 4	1.337	(0.823–2.172)	0.242
**Ulceration**			
Absent	1 (base)		
Present	1.767	(0.734–4.252)	0.204
**LVI**			
Absent	1 (base)		
Present	1.764	(1.103–2.819)	**0.018**
**Mitotic rate**			
0–10	1 (base)		
> 10	1.257	(0.755–2.095)	0.381
**Regression**			
Absent	1 (base)		
Present	0.773	(0.452–1.322)	0.348
**TIL**			
Absent	1 (base)		
Present, non-brisk	0.882	(0.476–1.633)	0.689
Present, brisk	0.969	(0.365–2.576)	0.950
**Histology**			
Unspecified/other	1 (base)		
Nodular	0.670	(0.314–1.428)	0.299
Superficial spreading	0.705	(0.334–1.491)	0.360
Acral	0.712	(0.182–2.793)	0.627
Desmoplastic	0.929	(0.261–3.300)	0.909

*HR* hazard ratio, *CI* confidence interval, *LVI* tumor-infiltrating lymphocyte, *TIL* tumor-infiltrating lymphocyte

### Subset Analysis of Recurrence-Free Survival by Receipt of Adjuvant Therapy

Of the patients who received adjuvant therapy, 12 (48%) experienced a recurrence versus 89 (44%) of those who did not receive adjuvant therapy (*p* = 0.678). Additionally, 2-year RFS for the adjuvant therapy subgroup was 67% (95% CI 45–82%) versus 69% (95% CI 62–75%) for the patients who did not receive adjuvant therapy (*p* = 0.962). Notably, however, the patients who received adjuvant therapy were more likely to have a positive SLN (76% vs. 23%; *p* < 0.001) and the presence of LVI (45% vs. 24%; *p* = 0.035). In a multivariable Cox proportional hazards model for recurrence that controlled for all clinicopathologic factors as previously described in Table 4 as well as SLN positivity status, receipt of adjuvant therapy was associated with significantly reduced hazard for recurrence (HR 0.42; 95% CI 0.18–0.95; *p* = 0.037).

### Subset Analysis for Recurrence Stratified by Era (2016–2019 vs. 2009–2015)

In the Cox proportional hazards analysis for recurrence including treatment era as a covariate, LVI remained significantly associated with recurrence (HR 1.88; 95% CI 1.16–3.04; *p* = 0.010). Additionally, treatment era (2016–2019 vs. 2009–2015) had an HR of 0.67 for recurrence, but this did not meet the threshold for statistical significance (95% CI 0.43–1.07; *p* = 0.093). The remaining clinicopathologic factors were not significantly associated with recurrence in this analysis.

Within the 2016–2019 cohort, LVI was notable for an HR of 1.93 (95% CI 0.85–4.39; *p* = 0.117) for recurrence. In the 2009–2015 cohort, LVI had an HR of 2.63 (95% CI 1.35–5.11; *p* = 0.004) for recurrence. Additionally, the presence of regression was associated with a reduced HR for recurrence (0.39, 95% CI 0.19–0.81; *p* = 0.012). The remaining clinicopathologic factors were not significantly associated with recurrence in either cohort subgroup.

## Discussion

This study aimed to contextualize patterns of recurrence and survival outcomes in clinical stage IIB/IIC melanoma for selection of patients for neoadjuvant immunotherapy, as to our knowledge, no studies have examined recurrence patterns in this patient population without microstaging data via SLNB. Although KEYNOTE-716 and Checkmate-76K have demonstrated the RFS benefit of adjuvant PD-1 blockade for patients with IIB/IIC melanoma,^8–10^ the inclusion criteria for these studies were based on pathologic staging after SLNB. In contrast, our study examined a real-world, clinically staged IIB/IIC cohort before nodal staging, which differed from the treatment/control arms of the aforementioned trials. This distinction is particularly relevant because clinical trials such as NCT03757689 currently seek to evaluate immune checkpoint blockade in this pre-surgical setting, for which nodal status is not available.^15^ Additionally, our study, with a median follow-up time longer than 5 years, offers valuable insight into pre-surgical factors associated with recurrence.

In our study, similar to prior studies examining recurrence patterns in stage II melanoma, the greatest risk of recurrence was within the first 3 years after surgical resection.^16,21,22^ The majority of the recurrences were within 2 years (65%), with 94% of the recurrences by 5 years. The median time to recurrence was 15.3 months, and the estimated 2-year RFS was 69%.

To compare recurrence rates of clinical stage IIB/IIC melanoma with those of prior studies, the recurrence rates for pathologic stage IIB/IIC/IIIC melanoma must be used to achieve an accurate estimate, as a portion of clinical stage IIB/IIC patients will be upstaged to IIIC disease. Weighted average estimation from prior studies evaluating recurrence for AJCC eighth-edition pathologic stage IIB/IIC/IIIC disease resulted in an approximate 2-year RFS of 60–65%, in line with our finding of 69%.^8–10,21,24^ In addition, preliminary data reported from NCT03757689 presented at the American Society of Clinical Oncology 2025 conference showed a 2-year RFS of 84% for clinical IIB/IIC patients treated with neoadjuvant-adjuvant pembrolizumab,^25^ a comparatively higher rate than for the cohort in the current study.

Varey et al.^26^ recently published a predictive model estimating RFS and OS in stage II melanoma. Whereas LVI was the only pre-surgical pathologic factor associated with recurrence in our cohort, Varey et al.^26^ found multiple factors that significantly predicted recurrence. Notably however, our study differed from their model, which spanned a temporal range of 1986 to 2022, with 63% of the patients not undergoing SLNB. Additionally, their cohort also included stage IIA melanoma, and predictive modeling for OS and RFS was reported for validated low-, intermediate-, and high-risk groups. In contrast, we report real-world outcomes data specifically for clinically staged IIB/IIC melanoma patients, with 97% undergoing SLNB. Given the aforementioned cohort and analysis differences, direct comparison with their nomogram is challenging, and the larger cohort in their nomogram likely has greater statistical power inherently to detect smaller differences.

Careful patient selection for immunotherapy, particularly in the neoadjuvant setting, is important due to the risk of potential immune-related adverse events (irAEs) as well as possible delay of surgery. The presence of LVI was significantly associated with recurrence in our cohort in the multivariable analysis. Evaluation of pre-surgical pathologic criteria such as LVI may offer insight into patients who are likely to experience recurrence and thus may be more likely to benefit from initiation of immunotherapy.

Importantly, as demonstrated in Fig. 3, the KM curves for RFS stratified by LVI begin to differentiate approximately 2 years after treatment and differ significantly at 3–5 years. This is a relevant consideration for future trials if the primary outcome is RFS at 3 years and beyond. Furthermore, when era effects were taken into account, LVI was not significantly associated with recurrence in the 2016–2019 cohort (HR, 1.93; *p* = 0.117), whereas in the 2009–2015 cohort, the HR was 2.63 (*p* = 0.004). Additionally, regression emerged as significantly associated with reduced risk of recurrence in the 2009–2015 cohort. This could have been due to the introduction of more effective adjuvant immunotherapy after 2015, potentially obscuring the impact of prognostic factors in the 2016–2019 era, with the caveat of reduced sample size limiting statistical power in this analysis.

Additionally, evaluation of on-treatment biospecimens also may give information on efficacy of neoadjuvant immunotherapy, as prior studies have shown robust biologic signals for systemic immune cell activation and tumor pathologic response.^27–30^ Personalization of selection for neoadjuvant/adjuvant therapy based on pathologic and on-treatment biologic criteria may help to optimize patient selection and outcomes. Data from NCT03757689 will offer further insight into subgroup pre-surgical pathologic/biologic characteristics of clinical stage IIB/IIC melanoma patients who may derive greater benefit from neoadjuvant immunotherapy.

The limitations of this study include its retrospective design, which can add inherent biases in patient selection. The study also may have had confounders that could not be excluded without true randomization. Additionally, 97% of the patients in our cohort underwent SLNB, which is known to reduce recurrence risk. Thus, any interpretations from this data cannot necessarily be applied to patients who do not undergo SLNB. Furthermore, this study was a single-institution investigation with a cohort of patients that had presented at or had been referred to an academic center, which may have introduced bias in the complexity of disease seen as well as the availability of treatments offered to this cohort for the management of their melanoma.

In this single-institution retrospective cohort study over 14 years, we found a 2-year RFS of 69%, as well as a significant association of pre-surgical LVI with recurrence in patients with clinical stage IIB/IIC melanoma. These data can help guide clinicians/researchers in the design and assessment of future studies evaluating neoadjuvant therapy for clinical stage IIB/IIC melanoma, as well as in optimization of patient selection.
